# Surgical Site Infection Following Surgery for Spine Trauma

**DOI:** 10.3390/jcm15083109

**Published:** 2026-04-19

**Authors:** Matthias Zolda-Neugebauer, Georgios Gkourlias, Ulrike Wittig, Arastoo Nia, Kambiz Sarahrudi

**Affiliations:** 1 Department of Orthopedics and Trauma Surgery, University Hospital Wiener Neustadt, 2700 Wiener Neustadt, Austria; georgios.gkourlias@wienerneustadt.lknoe.at (G.G.); ulrike.wittig@wienerneustadt.lknoe.at (U.W.); kambiz.sarahrudi@wienerneustadt.lknoe.at (K.S.); 2 Department of Orthopedics and Trauma Surgery, University Hospital Neunkirchen, 2620 Neunkirchen, Austria

**Keywords:** spine, trauma, orthopedics, vertebrae, infection, risk factor, surgery, instrumentation

## Abstract

**Background/Objectives**: Traumatic spinal fractures are common injuries, and a proportion of these cases require surgical stabilization using various operative systems. This study aimed to analyze the epidemiology of surgical site infections (SSIs) following exclusively trauma-related spinal surgery and to identify potential risk factors for their occurrence, as there is a lack of studies focusing on non-elective trauma-related spinal surgeries and SSI in the literature. **Methods**: This retrospective single-center analysis examined 710 patients with traumatic spinal injuries treated surgically between 2012 and 2022 at the Level I Trauma Center at the Department of Orthopedics and Trauma Surgery of the University Hospital Wiener Neustadt, Austria. To investigate SSI risk factors, comparative statistical analyses and logistic regression were used, with a level of statistical significance of α = 0.05. **Results**: In total, 28 cases (with an incidence of 3.94%) developed SSI, and these cases were characterized by a significantly higher body weight/BMI, longer operative times, and more stabilized segments and implanted hardware. They were also more likely to have undergone open surgery, laminectomy in combination with dorsal stabilization, intensive care treatment, or to present with neurological deficits or ankylosing spondylitis. SSIs occurred most frequently in the thoracolumbar and cervicothoracic junctions, and were predominantly caused by *Staphylococcus epidermidis*, *Staphylococcus aureus*, and *Cutibacterium acnes*. As independent risk factors, a higher BMI (OR = 1.188) and the use of cross-connectors (OR = 4.948) were identified, whereas other initially significant variables did not remain significant after adjustment. **Conclusions**: There are surgery-related and potentially modifiable variables and non-modifiable patient-related risk factors for the occurrence of SSI. Patients with SSIs stayed an average of 25.3 days in hospital and had a mortality rate of 17.9%.

## 1. Introduction

### 1.1. Spinal Fractures

Traumatic fractures of the spine are common injuries in traumatology and account for 48.9% of all spinal fractures [1].

A proportion of spinal fractures are treated surgically. The decision between surgical and conservative treatment is based on various criteria [2,3,4,5,6,7].

Surgically, the approach can be either dorsal or ventral, using minimally invasive or open techniques [8]. For stabilization, implants such as screws, plates, rods, or cross-connectors are utilized, with one or multiple segments potentially being fixed [9]. In addition, procedures such as vertebroplasty, kyphoplasty, laminectomy, or discectomy may be performed to support healing [8].

### 1.2. Surgical Site Infections (SSIs)

Surgical site infections (SSIs) occur following spinal surgery and complicate the healing process. The Centers for Disease Control and Prevention have defined the term surgical site infection, and distinguish between superficial, deep and organ/space infections based on clearly defined diagnostic criteria [10].

Their incidence is higher in trauma-related procedures than in elective surgeries. Ogihara et al. reported a rate of 3.2% [11,12], and in 2009, De Lissovoy et al. demonstrated that SSIs prolong hospital stays by an average of 9.7 days and lead to additional costs of USD 20,842 [13].

Potential sources of infection include the patient’s own skin, the surgical environment, and the operating team [14,15,16]. Common causative pathogens include *MRSA*, *MSSA*, *Enterobacter*, *coagulase-negative Staphylococcus*, *Enterococcus faecalis*, *Pseudomonas aeruginosa*, and *Staphylococcus epidermidis*, many of which are part of the normal skin flora [11,12,17,18].

Meta-analyses have identified diabetes mellitus, hypertension, obesity, surgical approach, duration of surgery, location, and number of stabilized segments as risk factors [19,20]. Spinal cord injuries, age, higher ASA scores and intensive care unit stays also have been associated with an increased risk of SSI [11,12,21,22].

The treatment of SSI is complex, as standardized therapeutic protocols are often lacking. Typically, management includes systemic antibiotic therapy, surgical debridement with possible implant removal, and appropriate wound care [23]. For prevention, the Robert Koch Institute recommends pre-, peri-, and postoperative measures, as well as minimizing modifiable risk factors [24].

The objective of this retrospective study was to investigate exclusively trauma-related spinal surgeries, as there is a lack of studies focusing on non-elective spinal surgeries and SSI. The aim was to present the epidemiology of SSI at the Level I Trauma Center at the Department of Orthopedics and Trauma Surgery of the University Hospital Wiener Neustadt in Austria and to identify possible risk factors.

## 2. Materials and Methods

**Figure 1:**
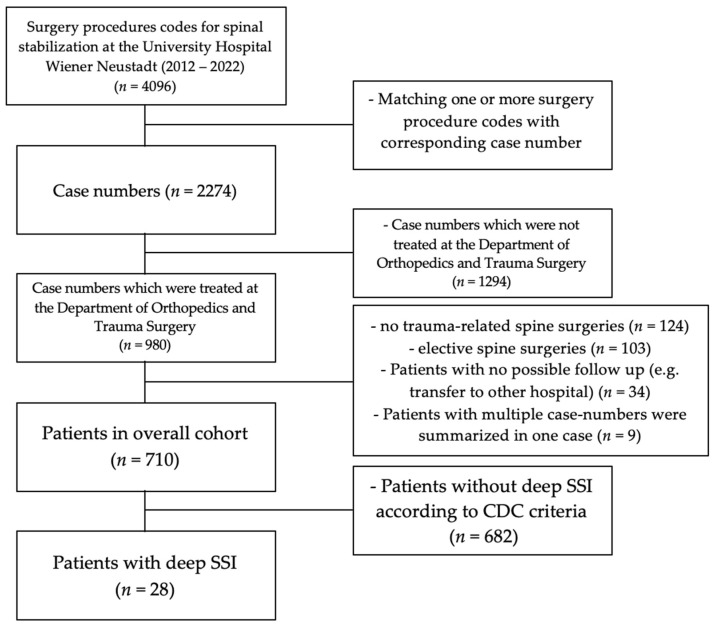
The identification process of the overall cohort and the patients with a deep SSI. CDC = Centers for Disease Control and Prevention; SSI = Surgical site infection.

The study was approved by the Ethics Committee of the Government of Lower Austria and performed in accordance with the ethical standards of the Declaration of Helsinki. For data collection, the hospital’s internal procedural codes were used to identify patients treated between 2012 and 2022. In total, 4096 procedural codes were identified across all hospital departments. These codes were subsequently assigned to their respective case numbers, as many case numbers included multiple procedures. This process resulted in a total of 2274 distinct case numbers. Subsequently, 980 case numbers were filtered for patients treated in the Department of Trauma Surgery. For these cases, the mechanism of injury and hospital stay were recorded. Of the 710 patients included in the study cohort, 28 developed a deep surgical site infection and formed the subgroup for detailed analysis. The identification process is shown in Figure 1.

Inclusion criteria comprised patients of both sexes with traumatic spinal injuries treated operatively with spinal implants at the University Hospital Wiener Neustadt, without age restrictions.

Inclusion in the specific cohort required the occurrence of a deep surgical site infection diagnosed according to CDC criteria, with a maximum follow-up of 12-month after the initial SSI diagnosis. The documentation of the clinical presentation, diagnostic findings (e.g., microbiological findings), and inpatient and outpatient treatments (e.g., antibiotic therapy, reoperation) during this 12-month period were collected and analyzed.

Exclusion criteria included elective surgeries, no trauma-related spine surgeries (osteoporotic or pathological fractures), procedures without implants and cases lacking follow-up data. Only deep incisional SSIs were analyzed in detail.

In addition to the epidemiological evaluation, a risk factor analysis was performed. For this purpose, the group of patients with a surgical site infection (SSI; *n* = 28) were compared with patients without a SSI (*n* = 682). The *t*-test or Mann–Whitney U test was applied for continuous variables, and the chi-square test or Fisher’s exact test were applied for categorical variables. The level of statistical significance was set at α = 0.05 for all tests, and two-tailed significance testing was used. Multivariable logistic regression was performed with SSI status (yes/no) as the dependent variable. Given that only 28 SSI events occurred, the number of predictors in the multivariable model was restricted to reduce the risk of overfitting. Statistical analyses were conducted using IBM SPSS Statistics 29, version 29.

During the preparation of this study, the authors used ChatGPT 3.5 for the translation of background information and support in interpreting statistical results. The authors have reviewed and edited the output and take full responsibility for the content of this publication.

## 3. Results

### 3.1. Epidemiology

A total of 28 deep surgical site infections (SSI) occurred among 710 operated patients, corresponding to an incidence of 3.94%. As shown in Table 1, men and women were equally represented in the total cohort and in the non-SSI group. However, SSI occurred more frequently in men. The median age of all patients was 66 years (range, 13–95 years), with those who developed an SSI tending to be older. There was a statistically significant association between high body weight, BMI and SSI occurrence (*p* < 0.001). The ASA scores did not differ between groups. Notably, patients with SSI had approximately twice the length of hospital stay as others, and their mortality rate was markedly higher at 17.9%.

Among all 710 patients, hypertension was the most common comorbidity (329 cases, 46.3%), followed by heart failure (100 cases, 14.1%), diabetes mellitus (93 cases, 13.1%), coronary artery disease (36 cases, 5.1%), and ankylosing spondylitis (34 cases, 4.8%). In the SSI group, 18 patients (64.3%) had hypertension, 7 (25%) heart failure, 5 (17.9%) diabetes mellitus, and 4 (14.3%) ankylosing spondylitis.

The most frequent trauma mechanisms were mechanical falls (325 cases, 46%), traffic accidents (127, 18%), falls from height (104, 15%), and sports injuries (88, 12%). A total of 10 patients (1%) sustained injuries from suicide attempts, and 56 cases (8%) could not be classified. Among the 28 SSI patients, 14 (50%) had mechanical falls, 5 (17.9%) traffic accidents, 3 (10.7%) falls from height, and 1 each (3.6%) sustained sports- or suicide-related injuries; 4 cases (14.3%) remained unclassified.

Neurological deficits were initially observed in 101 patients (14.8%) in the group without SSI, but in 12 patients (42.9%) in the group who later developed SSI. The most commonly affected spinal regions were the thoracolumbar (Th11–L2) and cervicothoracic (C6–Th2) junctions. In the SSI group, Th1 was the most frequently fractured vertebrae, occurring in 8 of 28 patients (28.6%), followed by Th12 (*n* = 5; 17.9%) and C6 (*n* = 4; 14.3%). The first lumbar vertebra (L1), which was the most frequently fractured vertebra in the overall cohort, was affected in three cases (10.7%) in the SSI group.

SSI occurred most often in the thoracic spine (37.9%), followed by the lumbar spine (31%), cervical spine (27.6%), and sacrum (3.4%). Dorsal stabilization was by far the most frequently used procedure (576 cases, 84.5%), although a pure dorsal stabilization was performed in 152 cases (21.4%). All vertebroplasties and kyphoplasties were performed in combination with dorsal stabilizations. All procedures of laminectomy were also combined with dorsal stabilizations. In many cases, multiple surgical techniques were used in combination. Other procedures were used consistently at lower frequencies. Among SSI patients, dorsal stabilization was performed in 27 cases (96.4%), while laminectomy was used more frequently (11 cases, 39.3%).

As shown in Table 2, the duration of surgery, number of implanted screws, and number of fused segments were significantly associated with the occurrence of SSI (*p* < 0.001). Patients with SSI had, on average, 1.5 more fused segments, and their operations lasted approximately three hours longer. On average, they received more screws, plates, and cross-connectors. In total, 18 patients received cross-connectors, of whom only one patient received two, while the remainder received one.

Of 20 microbiological samples, *Staphylococcus epidermidis* was detected in 10 cases (50%), including 3 methicillin-resistant strains (15%; *MRSE*). *Staphylococcus aureus* was identified in nine cases (45%), five (25%) of which were *MRSA*. *Cutibacterium acnes* was present in five samples, and *Enterococcus faecalis* in four (20%). Other bacterial species appeared only once.

In most cases (20 patients, 71.4%), treatment of SSI required one or more revision surgeries in addition to antibiotic therapy. Implant removal was necessary in four patients (14.3%). The mean duration of deep SSI was 97.6 days (SD, 247).

### 3.2. Risk Factor Analysis

Table 1 and Table 2 also present the analysis of continuous and ordinal variables. Significant differences were found between the SSI and non-SSI groups for body weight/BMI, number of stabilized segments, duration of surgery, and number of screws and cross-connectors used.

Table 3 presents the analysis of categorical variables. SSI were significantly associated with the presence of neurological deficits at admission, intensive care unit stay, open surgical approach, laminectomy, and ankylosing spondylitis. Significant correlations were also found for surgical approach and kyphoplasty; however, it should be noted that all infections occurred after dorsal access. Because two-tailed significance testing was used, kyphoplasty, in combination with dorsal stabilization, also showed a positive association.

The logistic regression analysis, comparing the SSI group with the non-SSI group, revealed a significant positive association for BMI (regression coefficient (B) = 0.173; *p* = 0.033; odds ratio (OR) = 1.188) and for the number of cross-connectors used (B = 1.599; *p* = 0.041; OR = 4.948). This indicates that for each one-unit increase in BMI, the odds of developing an SSI increase by approximately 19%. The use of cross-connectors was associated with an almost fivefold-higher risk of SSI.

The surgical approach also showed a significant association (combined approach; B = −2.848; *p* = 0.026; OR = 0.058), initially suggesting that the combined surgical approaches were, in fact, associated with a lower risk of infection. The observed protective effect of the combined approach should be interpreted with caution, as it is based on a very small number of cases and is associated with a wide confidence interval.

The results are shown in Table 4.

All other initially significant variables, including weight/BMI, duration of surgery, kyphoplasty, ankylosing spondylitis, neurological symptoms, laminectomy in combination with dorsal stabilization, intensive care unit stay, number of fused segments, and number of screws, did not demonstrate a significant effect on SSI occurrence.

The constant of the model (B = −5.051; *p* = 0.049; OR = 0.006) indicates that, in the absence of the significant risk factors, the baseline probability of infection is very low (odds = 0.006).

In summary, this analysis identified BMI and the number of cross-connectors as significant risk factors for the development of surgical site infection following trauma-related spinal surgery.

## 4. Discussion

The current literature provides limited comparative data focusing exclusively on the occurrence of surgical site infections (SSIs) after spinal trauma. In the present study, the incidence of SSI was 3.94%, which closely aligns with the findings of Ogihara et al., who reported an incidence of 3.2% in 623 similarly trauma-related spinal fixations [12].

In our data, SSIs were more frequently observed in men than in women, although this difference was not statistically significant, a finding consistent with the meta-analyses by Zhang et al. and Peng et al. [19,20]. Although older age has been described as a risk factor for SSI in several studies, no significant association was observed in our analysis [12,21,22]. In accordance with the literature, mechanical falls represented by far the most common mechanism of spinal injury.

Our data did not identify hypertension and diabetes mellitus as risk factors for SSI, in contrast to the reported comorbidities by the meta-analyses of Zhang and Peng et al. [19,20]. A significant difference was found between groups with respect to body weight and BMI. The risk analysis demonstrated a 19% increase in the odds of SSI for each unit increase in BMI, although it should be noted that the statistical analysis may be subject to overfitting of the results. Peng et al. similarly reported a significant risk of SSI in obese patients [20]. For the first time, our data indicate a potential association between SSI and ankylosing spondylitis, for which no comparable data are currently available in the literature. Ogihara et al. and Rechtine et al. described a higher SSI risk in patients with initial neurological symptoms, which was in contrast to our data [12,22]. Another discrepancy between the reported literature and our study was the described association between SSI and a higher ASA score, which was not supported by our findings [12]. For deep SSI, Zhang et al. found a significantly higher risk in cervical and lumbar regions compared to the thoracic spine [19]. In contrast, most infections in our data occurred in the thoracic region, particularly at its transitional levels at the thoracolumbar and cervicothoracic junctions.

Another important finding of the present study is that longer surgical duration, multi-segment dorsal stabilization, and increased implant use may contribute to a higher SSI risk. Lonjon et al. and Zhang et al. also found a significant association with the number of stabilized segments [19,21]. Ultimately, however, only the use of cross-connectors significantly increased the risk by nearly fivefold, although potential overfitting must be considered. All infections developed following dorsal approaches, which were also the most frequently performed. Interestingly, a combined ventrodorsal approach was associated with a potential reduction in SSI risk, although it should be noted that this result may be influenced by the low number of combined ventrodorsal approaches and the wide confidence interval. This result should be interpreted with caution, and may reflect selection bias and the limited statistical power of the model. The laminectomy, in combination with dorsal stabilization, initially showed a significant result; however, no correlation was found in the final analysis. This is in line with other parameters that prolong the surgery duration as noted above. Furthermore, ICU stay appeared to be potentially associated with SSI, but without an actual increase in risk, in contrast to the findings of Blam et al. [11].

This observation may be interpreted in light of recent evidence suggesting that the surgical strategy and approach play a critical role in postoperative complication profiles. Todeschi et al. and Canseco et al. also highlight that not only the anatomical region, but also the choice of surgical technique may substantially influence the risk of SSI and other complications [25,26].

On average, infections developed 18.6 days postoperatively, which is consistent with the timeframe described in the literature [10]. The mean duration of deep SSI was 97.6 days, although no comparable reference values were found.

The most frequently detected microbiological cultures were *Staphylococcus epidermidis*, *Staphylococcus aureus*, and *Cutibacterium acnes*, consistent with the findings of Guarch-Pérez et al., who described these organisms as common components of the normal skin flora and frequent SSI pathogens [17]. As noted in previous reports, treatment protocols for deep SSI varied considerably, with no standardized therapeutic approach identified [23].

According to De Lissovoy et al., SSI prolongs hospitalization by an average of 9.7 days; in our study, patients stayed 25.3 days longer on average [13]. The mortality rate among SSI patients was notably high at 17.9%, although no comparative data were available.

## 5. Limitations

A major limitation of this study is the unequal group sizes, which may have introduced statistical bias. The low number of patients with SSI (*n* = 28), compared with 682 patients without SSI, represents a potentially skewed distribution. The use of logistic regression, including only variables that were initially significant (*n* = 12) between the SSI and non-SSI groups, may have resulted in overfitting due to a very low events-per-variable (EPV) ratio and limited statistical power. Furthermore, the literature used for the identification and evaluation of risk factors often presented inconsistent findings, making direct comparison challenging. Several referenced studies also included non-traumatic spinal surgeries or procedures from other disciplines, thereby limiting comparability.

## 6. Conclusions

In this retrospective analysis of 710 patients, the incidence of SSI was 3.94%. Infections developed on average 18.6 days postoperatively and persisted for approximately 97.6 days, resulting in a mean prolongation of hospital stay of 25.3 days. The most frequently isolated pathogens were the skin commensals *Staphylococcus epidermidis*, *Staphylococcus aureus*, and *Cutibacterium acnes*.

Among surgery-related variables, potential modifiable risk factors included the number of stabilized segments, an open surgical approach, the number of screws and cross-connectors, and the performance of laminectomy. Non-modifiable risk factors included body weight/BMI, initial neurological symptoms, intensive care unit stay, and ankylosing spondylitis. Another important finding of this study is the fact that longer surgery duration can lead to significantly higher infection rates.

Moreover, a significantly increased risk of SSI was found for higher BMI (OR = 1.188) and the use of cross-connectors (OR = 4.948), corresponding to a 19% increase in odds per BMI unit and nearly a fivefold increase when cross-connectors were used. The mortality rate among SSI patients was 17.9%.

In summary, our results strongly indicate that longer operations, as well as non-minimally invasive procedures, may increase the risk of infection, and represent factors that are potentially modifiable.

Future research should focus exclusively on trauma-related spinal surgeries to enable more precise identification and quantification of risk factors for surgical site infections.

## Figures and Tables

**Table 1:** The patient-related data in the studied groups and significant differences for the continuous or ordinal variables between the SSI group and the rest.

Variable	Overall Cohort (*n* = 710)	Patients Without SSI (*n* = 682)	Patients with SSI (*n* = 28)	*p*-Value
Female, n (%)	332 (46.8)	322 (47.2)	10 (35.7)	0.252
Male, n (%)	378 (53.2)	360 (52.8)	18 (64.3)	0.252
Age (mean ± SD)	61.6 (20.2)	61.4 (20.3)	68 (16)	0.119
Weight, kg (mean ± SD)	77.4 (16.7)	76.9 (16.5)	88.1 (19)	<0.001
BMI (mean ± SD)	26.4 (4.8)	26.4 (6.4)	30 (6)	<0.001
ASA score (mean ± SD)	2.5 (0.8)	2.5 (0.8)	2.5 (1)	0.323
Hospital stay (days, mean ± SD)	19.1 (19)	18.1 (17.9)	43.4 (27)	/
Deceased, n (%)	28 (3.9)	23 (3.4)	5 (17.9)	/

SD = standard deviation.

**Table 2:** Surgical data groups and significant differences for the continuous or ordinal variables between the SSI group and the rest.

Variable	Overall Cohort (*n* = 710)	Patients Without SSI (*n* = 682)	Patients with SSI (*n* = 28)	*p*-Value
Time from injury to surgery (days, mean ± SD)	9.2 (12.8)	9.1 (12.9)	10.3 (12.3)	0.496
Number of stabilized segments (mean ± SD)	2.5 (1.4)	2.4 (1.3)	3.9 (2.3)	<0.001
Number of involved surgeons and nurses (mean ± SD)	3.1 (0.6)	3.1 (0.6)	3.3 (0.6)	0.147
Operative time (min, mean ± SD)	105.8 (67.7)	103.1 (67.3)	180.5 (105.5)	<0.001
Number of screws (mean ± SD)	5.3 (2.6)	5.2 (2.5)	8.2 (4.1)	<0.001
Number of longitudinal rods (mean ± SD)	1.7 (0.8)	1.7 (0.8)	1.9 (0.4)	0.143
Number of cross-connectors (mean ± SD)	0.03 (0.2)	0.02 (0.1)	0.21 (0.5)	<0.001
Number of plates (mean ± SD)	0.09 (0.3)	0.08 (0.3)	0.14 (0.4)	0.247

SD = standard deviation; min = minutes.

**Table 3:** Comparison of demographic, clinical, and surgical categorical parameters between the two groups and their association with SSI.

Variable	Patients Without SSI (*n* = 682)	Patients with SSI (*n* = 28)	*p*-Value
Gender			0.252
Female	322 (47.2)	10 (35.7)	
Male	360 (52.8)	18 (64.3)	
Surgical approach			<0.001
Dorsal	571 (83.7)	22 (78.6)	
Ventral	101 (14.9)	/	
Combined ventral/dorsal	10 (1.5)	6 (21.4)	
Surgical technique			<0.001
Minimally invasive	480 (70.4)	9 (32.1)	
Open	202 (29.6)	19 (67.9)	
Patients requiring intensive care	144 (21.1)	14 (50)	<0.001
Preoperative antibiotic prophylaxis	615 (90.2)	24 (85.7)	0.493
Neurological symptoms	101 (14.8)	12 (42.9)	<0.001
Only dorsal stabilization	147 (21.6)	5 (17.9)	0.812
DS + vertebroplasty	231 (33.9)	8 (28.6)	0.685
DS + kyphoplasty	143 (21)	1 (3.6)	0.028
DS + laminectomy	55 (8.1)	11 (39.3)	<0.001
Discectomy	47 (6.9)	3 (10.7)	0.439
Odontoid screw fixation	44 (6.5)	1 (3.6)	1
Ventral plating	67 (9.8)	5 (17.9)	0.191
C1/C2 fusion	26 (3.8)	1 (3.6)	1
Hypertension	311 (45.6)	18 (64.3)	0.056
Diabetes mellitus	88 (12.9)	5 (17.9)	0.397
Heart failure	93 (13.6)	7 (25)	0.098
Coronary artery disease	36 (5.3)	0 (0)	0.391
Ankylosing spondylitis	30 (4.4)	4 (14.3)	0.040
Cervical spine injury	153 (22.4)	10 (35.7)	0.110
Thoracic spine injury	251 (36.8)	12 (42.9)	0.552
Lumbar spine injury	314 (46)	9 (32.1)	0.177
Sacral injury	9 (1.3)	1 (3.6)	0.333

DS = dorsal stabilization.

**Table 4:** The increased and decreased risk of developing an SSI in the presence of the identified risk factors.

	OR	95% CI	*p*-Value
BMI	1.188	1.015–1.390	0.033
Combined approach	0.058	0.005–0.724	0.026
Cross-connectors	4.948	1.075–22.776	0.041

OR = odds ratio, CI = confidence interval.

## Data Availability

The data used in this study are not publicly available due to data protection regulations. No public links or similar access points are provided. The dataset was created solely for the purpose of scientific analysis.

## Abbreviations

The following abbreviations are used in this manuscript:

ASA	American Society of Anesthesiologists
BMI	Body mass index
*MRSA*	Methicillin-resistant *Staphylococcus aureus*
*MSSA*	Methicillin-sensitive *Staphylococcus aureus*
*MRSE*	Methicillin-resistant *Staphylococcus epidermidis*
SSI	Surgical site infection
SPSS	Statistical Package for the Social Sciences
SD	Standard deviation
n	Number
Th	Thoracic
C	Cervical
OR	Odds ratio
B	Regression coefficients
ICU	Intensive care unit
kg	Kilogram
L	Lumbar
e.g.,	Exempli gratia
EPV	Events per variable
CDC	Centers for Disease Control and Prevention
